# MGMT and MSH6 immunoexpression for functioning pituitary macroadenomas

**DOI:** 10.1007/s11102-017-0829-3

**Published:** 2017-09-12

**Authors:** Alexander S. G. Micko, Adelheid Wöhrer, Romana Höftberger, Greisa Vila, Christine Marosi, Engelbert Knosp, Stefan Wolfsberger

**Affiliations:** 1 Department of Neurosurgery, Medical University Vienna, Waehringer Guertel 18-20, 1097 Vienna, Austria; 2 Institute of Neurology, Medical University Vienna, Vienna, Austria; 3 Division of Endocrinology and Metabolism, Department of Internal Medicine III, Medical University Vienna, Vienna, Austria; 4 Division of Oncology, Department of Internal Medicine I, Medical University of Vienna, Vienna, Austria

**Keywords:** Aggressive pituitary adenoma, MGMT, MSH6, TMZ

## Abstract

**Purpose:**

Knowledge of biological behavior is crucial for clinical management of functioning pituitary macroadenomas. For recurrent cases unresponsive to standard treatment, temozolomide (TMZ) has been used as a therapeutic alternative. MGMT (O6-methyl-guanine-DNA methyltransferase) and MSH6 (mutS homolog 6) immunoexpression have been linked to the response to TMZ treatment and MGMT immunoexpression has been additionally linked to early recurrence of non-functioning pituitary adenomas. The aim of this study was to assess the prognostic value of MGMT and MSH6 immunoexpression for aggressive functioning pituitary adenomas.

**Methods:**

The study cohort comprised a single center series of 76 patients who underwent an operation for functioning pituitary macroadenoma. We retrospectively compared 38 patients with postoperative persistent or recurrent disease with another set of 38 patients who were in endocrine remission.

**Results:**

Low-to-moderate MGMT immunoexpression (<50%) was significantly more frequent in the group with persistent/recurrent disease than in cases of endocrine remission (66 vs. 21%, p < 0.001). Furthermore, adenomas with low-to-moderate MGMT immunoexpression were significantly more often recurrent (76 vs. 30%, p < 0.001) and invasive (64 vs. 28%, p = 0.002).

**Conclusion:**

In our series, low-to-moderate MGMT immunoexpression was the only marker that significantly correlated with surgical invasiveness and recurrence in functioning pituitary macroadenomas. Therefore, in the future, MGMT status may be considered an additional marker for understanding the biological behavior of pituitary adenomas.

## Introduction

Depending on their biological behavior, recurrent functioning pituitary macroadenomas may pose a significant treatment challenge. Certain of these adenomas, although histopathologically classified as benign, remain incurable despite multiple surgeries, medical treatment and radiation treatment [1, 2]. In an effort to identify tumors with a more aggressive biological behavior, a histopathological category intermediate between typical adenomas and pituitary carcinomas, termed “atypical” adenomas, was established by the WHO Classification of Tumours of Endocrine Organs in 2004. These constitute as many as 2.7–15% of all pituitary adenoma cases [3, 4]. Due to a wide spectrum of behaviors that are not entirely benign and can cause significant morbidity, this terminology is currently under re-evaluation and will be revised in an upcoming WHO edition [5].

Primarily a mainstay of treatment for patients with high-grade gliomas and advanced melanoma [6, 7], the alkylating agent temozolomide (TMZ) has also shown its effectiveness against malignant neuroendocrine tumors [8]. In the search for additional therapeutic agents for aggressive pituitary adenomas and carcinomas unresponsive to standard treatment, TMZ has been used.

A positive response to TMZ has been described to depend on the downregulation of the DNA repair enzyme MGMT [9]. Initial reports from pituitary adenomas confirmed this association of a low MGMT immunoexpression with a positive response to TMZ treatment [10–14]. With increasing patient numbers, cases with low MGMT immunoexpression that do not respond to TMZ treatment have been reported [15], corroborating that additional factors might influence the response to TMZ.

An additional marker for the response to TMZ therapy was reported to be MSH6, a DNA mismatch repair protein [16]. MSH6 deficiency in gliomas was associated with negative response to TMZ therapy independently of MGMT status [17]. In a first small series of aggressive pituitary adenomas and carcinomas retrospectively tested for MSH6 status, the positive response to TMZ treatment corresponded to a high immunoexpression of MSH6 [18]. However, in further studies a loss of MSH6 in the presence of low MGMT immunoexpression was associated with TMZ resistance [19, 20].

Although small case series evaluated the MGMT status prior to TMZ treatment in aggressive adenomas and pituitary carcinomas, a systematic evaluation of MGMT and MSH6 status in functioning pituitary macroadenomas has not been performed thus far. From our observation that low-to-moderate MGMT immunoexpression correlates with early recurrence in non-functioning pituitary adenomas [21], the aim of the current study was to investigate the status of MGMT and MSH6 immunoexpression in a consecutive series of recurrent functioning macroadenomas. Further, we compared our results to those from a control group of patients in remission to determine (1) the prognostic value of MGMT and MSH6 for aggressive biological adenoma behavior and (2) to predict a potential response to TMZ therapy.

## Methods

### Patients

The study cohort comprised a retrospective, single center series of 76 patients who underwent an operation for histopathologically verified clinically functioning pituitary macroadenoma between 1997 and 2014. All patients were operated on by expert pituitary surgeons and the goal was gross total tumor resection in every case.

The study group consisted of 38 patients (group PD); a combined group of 28 patients with postoperative persistent disease (due to invasiveness) and 10 patients with recurrent disease after surgery for functioning pituitary macroadenoma. The control group consisted of 38 patients in endocrine remission (group ER) operated on in the same time period who were matched by adenoma subtype, age at first surgery, follow-up and gender distribution.

This study was approved by the ethics committee (EC Nr: 1008/2014) and was performed in accordance with the principles of the Declaration of Helsinki. The tumor samples were evaluated with the consent of the patients for further histopathological examination.

Surgical cure was defined by endocrine remission (ER) with the following characteristics: morning serum cortisol level (<5 μg/dl) alone or combined with normal 24-h urinary free cortisol (UFC) level in adrenocorticotropic hormone (ACTH) producing adenomas [22]; suppressed growth hormone (GH) less than 0.4 ng/ml during oGTT (oral glucose tolerance test) or a random GH less than 1.0 ng/ml and normal IGF1 in GH producing adenomas [23]; normal prolactin (PRL) in PRL producing adenomas [24]; normal free T_4_ level in thyroid-stimulating (TSH) producing adenomas [25]; postoperatively controlled at outpatient clinic: 4 weeks, 3 months, 6 months and afterwards once yearly. Patients who, subsequent to surgery, developed abnormal concentration values during follow-up and required medical treatment were categorized as recurrent disease.

Postoperative persistent or recurrent disease (PD) was defined by elevated plasma cortisol or UFC level; elevated IGF1 and insufficient suppressed GH during oGTT or a random GH > 1.0 ng/ml; elevated PRL; elevated free T_4_ and TSH level; ±radiologically verified residual tumor postoperatively controlled at outpatient clinic: 4 weeks, 3 months, 6 months and afterwards dependent on the progress of tumor growth (at least once a year).

Tumor invasion was based on MR imaging as well as intraoperative signs of invasion into the cavernous sinus and/or surrounding structures.

### Histopathological examinations

We examined the molecular markers MGMT and MSH6 in addition to our standard histopathological workup including MIB-1, in all patients. If MIB-1 was >3%, the additional criteria of high immunoexpression of p53, mitoses >2/10 high-power fields (HPF) and nuclear pleomorphism within the specimen had to be fulfilled for identification of an atypical adenoma. Additionally, the clinicopathological classification proposed by Trouillas et al. [26] was included. Signs of invasion as well as two or more of the following proliferation markers were considered: MIB-1 > 3%, mitoses > 2/10 HPF, p53 positive (10 strongly positive nuclei/10 HPF).

### Tissue processing

All biopsy specimens were fixed in 4% neutral buffered formalin, routinely processed, embedded in paraffin, cut at 5 μm and stained with hematoxylin and eosin. Immunohistochemistry (IHC) was performed with a streptavidin-biotin-peroxidase complex method.

The following antibodies were used for staining with a Dako AutostainerPlus Link automated immunostainer: ACTH (DakoCytomation, Glostrup, Denmark; monoclonal; 1:1000), GH (Dako; polyclonal; 1:5000), PRL (Dako; polyclonal 1:4000), TSH (Biogenex, Sanraman, CA; monoclonal;1:500), luteinizing hormone (Dako; monoclonal; 1:50), follicle-stimulating hormone (Biogenex; 1:500), the alpha-subunit of glycoprotein hormone (Acris, San Diego, CA; monoclonal; 1:40 000), MIB-1 (Dako; monoclonal; 1:200) and p53 (Dako; monoclonal; 1:50). For pretreatment and visualization, the Envision FLEX Plus Dako kit was used according to the manufacturers recommendations.

IHC with mouse monoclonal antibodies for MGMT (Neomarkers, Fremont, CA; 1:50) and MSH6 (Cell Marque, Rocklin, CA; 1:250) was performed as follows: after deparaffinization, 3–5 mm thick sections underwent heat-induced epitope retrieval in citrate buffer, pH 6.0. Endogenous peroxidase activity was blocked with 3% hydrogen peroxide in methanol (10 min). After each following step, sections were washed with 0.01 M PBS (pH 7.4) 3 times. Tissue sections were incubated overnight at 4 °C with the primary antibody. The next day, sections were labelled with the appropriate secondary antibody, incubated with avidin-biotin-peroxidase, and visualized with a standard diaminobenzidine (DAB) detection kit (Dako Envision). Sections were then counterstained with Mayer’s hematoxylin. IHC for pan-cytokeratin was used to characterize densely and sparsely granulated GH adenomas.Positive control tissues consisted of paraffin-embedded sections of colon cancer for immunostaining of MGMT and MSH6. As a negative control, we used a non-relevant antibody of the same species (mouse) and of the same immunoglobulin isotype (IgG1).

### Assessment

The MIB-1 labelling index was evaluated manually as the percentage of immunopositive cells per 500 cells in the hotspot area. For MGMT, the specimens were divided into three groups: <10% (low), 10–50% (moderate) and >50% (high) immunopositive cells, as described previously [9, 27]. For MSH6, the specimens were classified into a four-tiered score: 0: immunonegative, 1: <10%, 2: 10–50%, or 3: >50% immunopositive cells. A score ≥ 2 (>10% immunopositive cells) was considered immunopositive [18].

The immunoreactivity of MGMT and MSH6, as well as the MIB-1 labelling index, was evaluated microscopically (Olympus BHS, Tokyo, Japan) by 3 observers (A.M., A.W. and R.H.) blinded to the extent of surgical resection. Only areas with highest immunoreactivity and minimal necrosis, fibrosis or artifacts were selected for evaluation.

### Statistical analysis

The data are presented as the means and ranges for continuous variables and as frequencies for categorical variables. To assess the difference in MGMT and MSH6 status between the two groups (group PD and group ER) χ^2^-test with Pearson’s correlation coefficient was performed. Differences in the MIB-1 index between the two groups were assessed by unpaired *t* tests. To assess potential prognostic variables of markers for invasiveness, we evaluated MIB-1, MGMT < 10, MGMT < 50 and MSH6 < 10 using a binary logistic regression analyses.

A p value <0.05 was considered significant. For statistical analyses SPSS® version 21.0 software (SPSS Inc., Chicago, IL, USA) was used.

## Results

### Patient characteristics

Our study cohort (group PD) consisted of 38 consecutive patients with functioning pituitary macroadenomas and postoperative persistent or recurrent disease. The control group (group ER) consisted of 38 patients with matched subtypes of functioning macroadenomas in postoperative endocrine remission.

Of these 76 patients, histological subtypes were composed of 8 ACTH adenomas (8 basophilic), 26 GH adenomas (17 acidophilic, 9 chromophobic), 40 PRL adenomas (20 chromophobic, 14 acidophilic, 6 both) and 2 TSH adenomas (2 basophilic). In the case of the 40 PRL adenomas, the indication for surgery was resistance to dopamine-agonist therapy (n = 10), rapid visual loss by mass effect due to cystic component and/or apoplexy (n = 19), and patient preference instead of medical treatment (n = 11). In the case of GH adenomas, all patients were naïve to sandostatin analogues prior to the operation. In total, 15 atypical adenomas were identified.

The variables between the groups were not significantly different except for size (p = < 0.001), invasiveness (p = < 0.001) and granulation pattern of GH adenomas (p = 0.04). For further patient characteristics, see Table 1.

**Table 1:** Patient Characteristics

	Group PD		Group ER		P
	n (range)	%	n (range)	%	
Number of patients	38		38		
Number of surgeries	1.2 (1–4)		1		
Follow-up (years)	7.9 (0.3–26)		5.1 (0.4–20)		NS
Time to 2nd surgery (years)	0.3–7		/		
Age at first surgery (years)	42 (7–80)		40 (16–73)		NS
Gender					
Male	18	47	15	39	NS
Female	20	53	23	61	NS
Size (mm)	25 (12–45)		18 (11–40)		<0.001
Approach at 1st surgery					
Transsphenoidal	38	100	38	100	
Invasiveness (radiological/surgical)	27	71	6	16	<0.001
MIB-1 (%)	3.4 (0.3–15.7)		3.5 (0.4–18.1)		NS
Atypical adenoma	7	18	8	21	NS
Histologic subtype					
ACTH (Cushing’s disease)	4	11	4	11	
GH (acromegaly)	13	34	13	34	
Densely granulated	6	(46)	11	(85)	0.04
Sparsely granulated	7	(54)	2	(15)	
PRL (prolactinoma)	20	53	20	53	
TSH	1	3	1	3	

### Assessment of MGMT and MSH6 immunoexpression

Surgical tumor samples were histopathologically analyzed for immunoexpression of MGMT, MSH6 and MIB-1. Immunohistopathological examination was possible in all 76 cases.

#### MGMT

The frequency of low MGMT immunoexpression (<10%) was 20/76 (26%) across all cases. Low MGMT immunoexpression was significantly more frequent in group PD 17/38 (45%) than in group ER 3/38 (8%; p < 0.001) (Table 2).

**Table 2:** Overview—MGMT and MSH6 expression in functioning pituitary macroadenomas

	Overall	Group PD	Group ER	P
	n (%)	n (%)	n (%)	
MGMT expression				
	76 (100)	38 (50)	38 (50)	NS
Low				
<10%	20 (26)	17 (45)	3 (8)	<0.001
10–50%	13 (17)	8 (21)	5 (13)	NS
0–50%^a^	33 (43)	25 (66)	8 (21)	<0.001
High				
>50%	43 (57)	13 (34)	30 (79)	<0.001
MSH6 expression				
	76 (100)	38 (50)	38 (50)	NS
Low				
0%	13 (17)	9 (24)	4 (10)	NS
1–10%	30 (39)	15 (39)	15 (40)	NS
0–10%^b^	43 (57)	24 (63)	19 (50)	NS
High				
>10%	33 (43)	14 (37)	19 (50)	NS

*NS* non significant (p > 0.05)

^a^Combining the groups <10% and 10–50%

^b^Combining the groups 0% and 1–10%

Tumors with low MGMT immunoexpression showed a significantly higher rate of recurrence (p < 0.001), but no significance for higher MIB-1 (p > 0.05), higher rate of invasiveness (p > 0.05), higher rate of atypia (WHO, p > 0.05; Trouillas Classification: 1a = 3/26; 1b = 9/17; 2a = 17/25; 2b = 4/8) or higher rate of positive MSH6 immunoexpression (p > 0.05) was found when compared to tumors with moderate-to-high MGMT (>10%) immunoexpression (Table 3).

**Table 3:** MGMT and MSH6 expression results

	MIB-1		Invasive		Recurrent		Atypical	
	mean	P	n/n	P	n/n	P	n/n	P
MGMT expression								
<10% (low)	3.7		12/20		17/20		5/20	
		NS		NS		< 0.001		NS
>10% (moderate-to-high)	3.4		21/56		21/56		10/56	
<50% (low-to-moderate)	3.2		21/33		25/33		8/33	
		NS		0.002		< 0.001		NS
>50% (high)	3.6		12/43		13/43		7/43	
MSH6 expression								
<10% (low)	2.8		23/43		24/43		7/43	
		NS		0.04		NS		NS
>10% (high)	4.3		10/33		14/33		8/33	

*NS* non-significant (p > 0.05)

The frequency of low-to-moderate MGMT immunoexpression (<50%) was 33/76 (43%) across all cases. Low-to-moderate MGMT immunoexpression was significantly more frequent in group PD 25/38 (66%) than in group ER 8/38 (21%; p < 0.001) (Table 2).

Tumors with low-to-moderate MGMT immunoexpression showed a significantly higher rate of invasiveness (p = 0.002) and recurrence (p < 0.001) but no significance for higher MIB-1 (p > 0.05), higher rate of atypia (WHO, p > 0.05; Trouillas Classification: 1a = 3/26; 1b = 9/17; 2a = 17/25; 2b = 4/8) or higher rate of positive MSH6 immunoexpression (p > 0.05) was found compared to tumours with high MGMT (>50%) immunoexpression (Table 3).

#### MSH6

The frequency of low MSH6 immunoexpression (<10%) was 43/76 (57%) across all cases. Low MSH6 immunoexpression was not more frequent in group PD 24/38 (63%) than in group ER 19/38 (50%; p > 0.05) (Table 2).

Tumors with low MSH6 immunoexpression showed a higher rate of invasiveness (p = 0.04), but no significantly higher MIB-1 (p > 0.05), higher rate of recurrence (p > 0.05) higher rate of atypia (WHO, P > 0.05; Trouillas Classification: 1a = 11/26; 1b = 9/17; 2a = 18/25; 2b = 5/8) or higher rate of low MGMT (p > 0.05) was found compared to tumors with high MSH6 (>10%) immunoexpression (Table 3).

### Logistic regression

We performed logistic regression analyses with invasiveness as the dependent variable. The only variable that remained independently significant for predicting invasiveness was low-to-moderate MGMT (<50%) immunoexpression (95% CI 1.43–24.86, OR 5.95, p = 0.014).

### Low MGMT with concurrent low/high MSH6 immunoexpression

We further analyzed the patient subgroup with low MGMT (<10%) and low MSH6 (<10%) immunoexpression. In total 12/76 (16%) cases fulfilled both criteria. Of these 12 patients (8 PRL, 3 GH and 1 TSH) 9/12 (75%) belonged to the group PD with a mean MIB-1 of 2.9. Three of these patients fulfilled the criteria of an atypical adenoma.

In the case of applying low MGMT (<10%) and high MSH6 (>10%) immunoexpression, 8/76 (11%) patients fulfilled both criteria. Of these 8 patients (7 PRL, 1 GH) all patients were in group PD with a mean MIB-1 of 4.9. Two of these patients fulfilled the criteria of an atypical adenoma.

### MGMT, MSH6 and MIB-1 immunoexpression after radiation and further surgery

In 11/38 of these cases (29%) from the PD group with multiple operations, standard medical treatment as well as radiation therapy, either by gamma-knife or external radiation (linear particle accelerator), had been performed. These 11 cases were identified as 4 ACTH, 6 GH and 1 PRL adenoma. The initial MIB-1 was 2.9 (0.3–5.7) at the time of the 1st operation and 7.1 (0.5–27.6) at the time of last operation, and only 1 GH adenoma was operated on one time and it did not reoccur after following gamma-knife. In all 6 GH (irrespectively of granulation pattern) and in 1 ACTH producing adenomas, a stable tumor mass was assessed at follow-up controls after radiation therapy. Furthermore, none of these patients necessitated any further surgery.

There were 4 cases (3 ACTH, 1 PRL) that did not respond to surgery and radiation, initial MGMT status changed in 3 of these cases (75%). In 2 ACTH cases MGMT status changed from 0–10% to 10–50%. In one case of PRL, MGMT status changed from >50% to <10%. In all 4 cases, MSH6 status did not change. In one of these 2 ACTH cases, evolution to a pituitary carcinoma occurred 7 years after the first operation. In this case, gamma-knife was performed 4 months after the initial operation.

### Aggressive pituitary adenomas and TMZ treatment

Three patients with aggressive functioning macroadenomas (1 PRL; 2 ACTH) were treated with TMZ at our department, after surgical intervention, medical treatment and radiation therapy were not possible any more. Each case had been discussed in an interdisciplinary board and received a clinical protocol consisting of oral TMZ, 200 mg m-2, 5 days every 28 days.

One patient (1 PRL) showed an initial TMZ response but tumor growth reoccurred while still receiving TMZ. MGMT status was <10%, and MSH6 was >10% before starting the treatment.

**Fig. 1:**
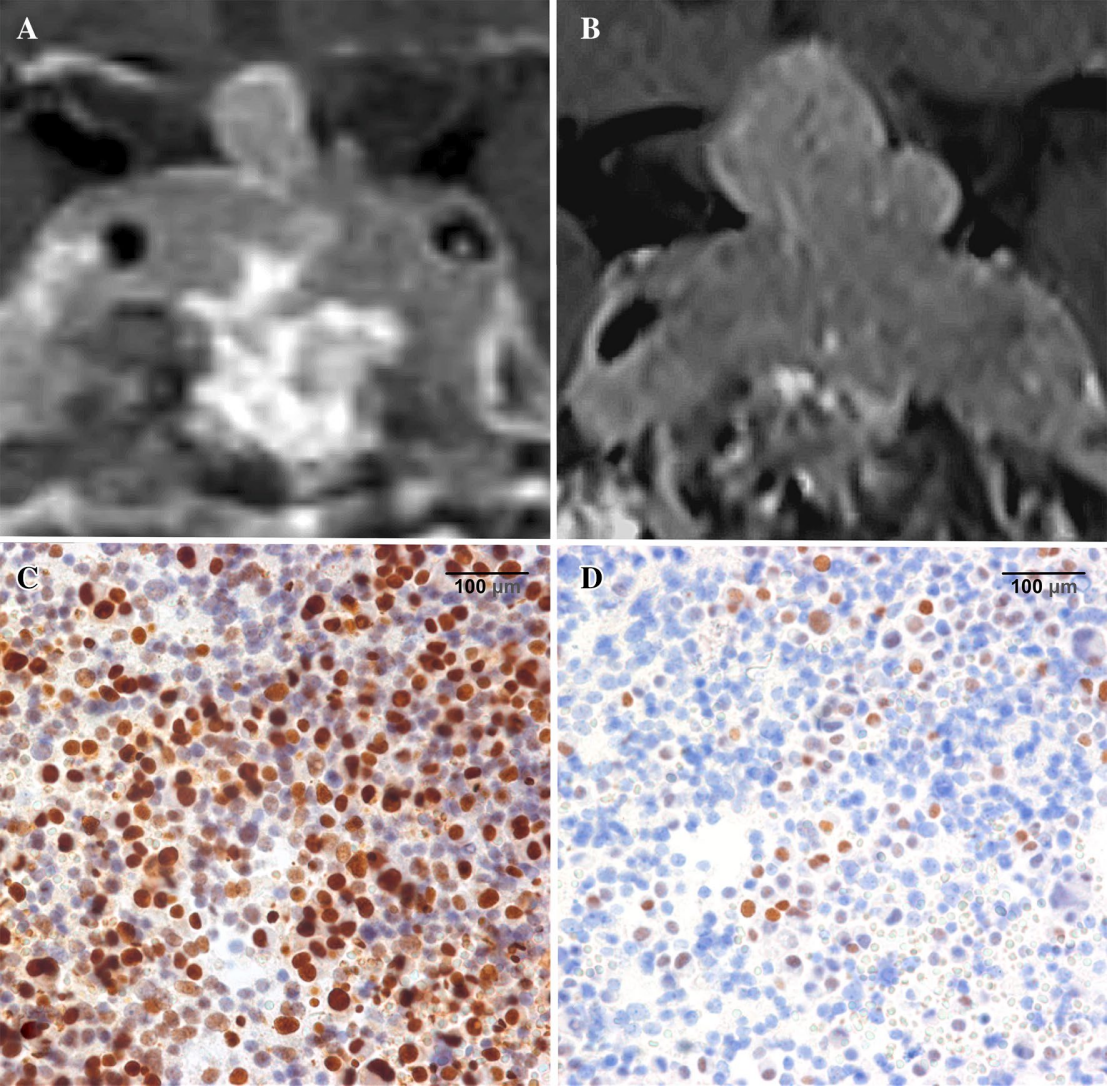
TMZ non-responder—Nelson tumor in a 55-year-old female patient. **a** Coronal MRI at the start of TMZ treatment shows recurrence of the invasive adenoma with high MIB-1 (13.8%) despite multimodal treatment (multiple transsphenoidal operations, 54 Gy external radiation, pasireotide therapy). **b** Coronal MRI 6 months after the first TMZ cycle; tumor regrowth occurred under therapy. Due to visual decline, TMZ was discontinued after 4 months and microsurgical partial resection of the tumor recurrence was performed by a subfrontal approach. **c** MGMT IHC sample from the last operation before TMZ treatment shows high MGMT immunoexpression (>50%) ×40 magnification. **d** MSH6 IHC sample from the last operation before TMZ treatment shows high MSH6 immunoexpression (10–50%) ×40 magnification

One patient with an ACTH tumor and Nelson’s syndrome showed progression during TMZ treatment. MGMT status was >50%, and MSH6 was >10% before starting the treatment. In this patient, MGMT as well as MSH6 status, were equal to the time of the first operation and did not change over the follow-up period (Fig. 1).

**Fig. 2:**
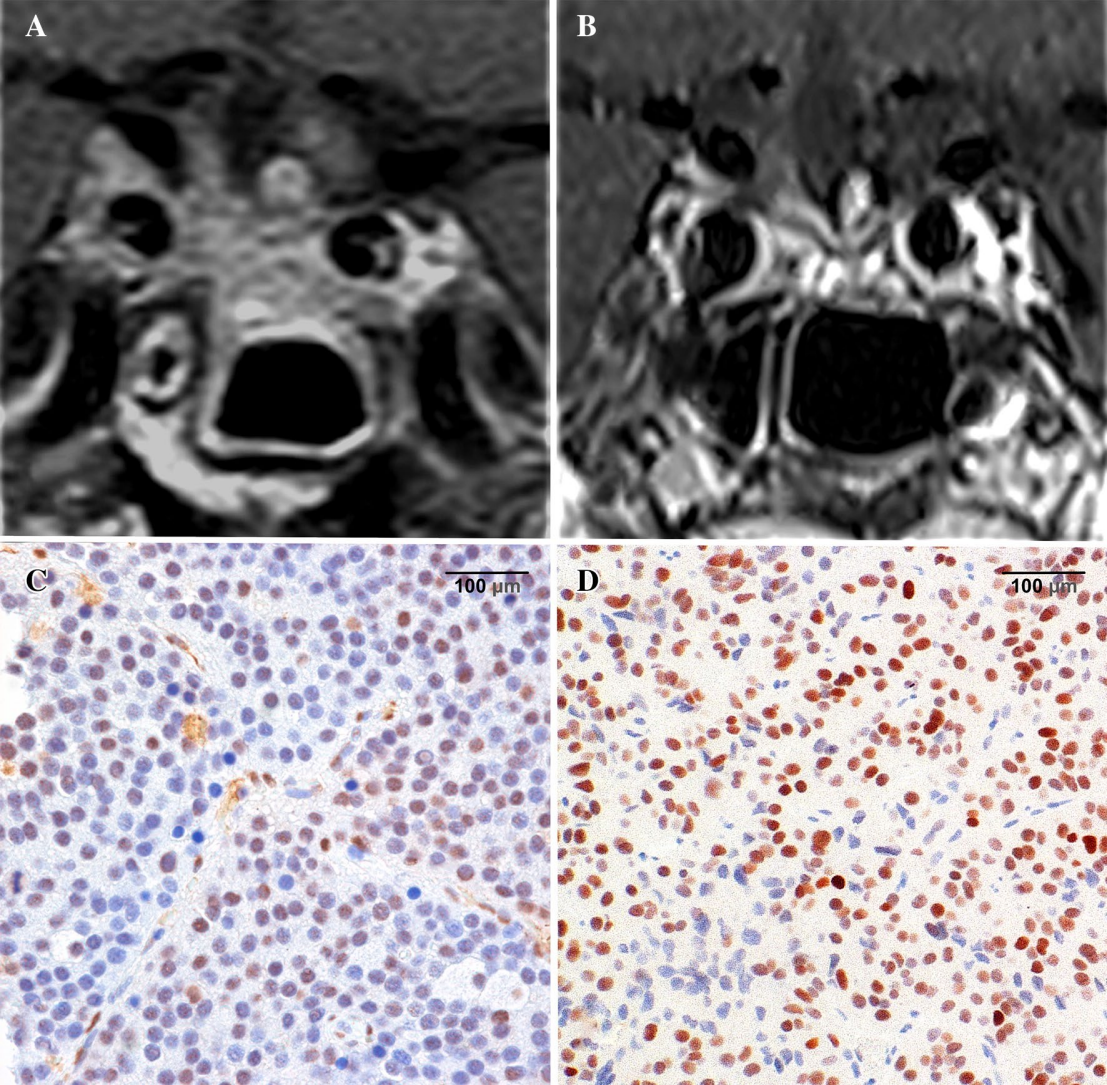
TMZ responder—invasive silent corticotroph adenoma subtype 1 in a 58-year-old male patient. **a** Coronal MRI at the start of TMZ treatment shows adenoma recurrence despite multimodal treatment (multiple transsphenoidal and subfrontal operations, pasireotide therapy). **b** Coronal MRI 6 months after the first TMZ cycle; although discontinued by the patient after 3 months due to side effects, a marked response to the TMZ treatment was found during follow-up and basal cortisol levels dropped to the normal range. **c** MGMT IHC sample from the last operation before TMZ treatment shows moderate MGMT immunoexpression (25–50%) ×40 magnification. **d** MSH6 IHC sample from the last operation before TMZ treatment shows high MSH6 immunoexpression (>50%) ×40 magnification

In the case of one ACTH adenoma (initially silent corticotroph adenoma subtype 1, with high cortisol levels at the time of recurrence), the tumor showed a response to treatment. MGMT status was 25–50%, and MSH6 was >10% before starting the treatment (Fig. 2).

## Discussion

Functioning pituitary macroadenomas that are progressive despite surgical, medical and radiation treatment may limit life expectancy by tumor mass effects and/or impact of hormone overproduction [28, 29]. To plan the frequency of radiological surveillance and additional treatment regimens, early detection of such aggressive adenoma behavior is therefore crucial.

In the present series of aggressive functioning macroadenomas we assessed the predictive value of the biological markers MGMT and MSH6. Low-to-moderate MGMT immunoexpression was significantly more common in patients with progressive disease than in patients in remission, whereas MSH6 did not differ between the groups.

Low-to-moderate MGMT immunoexpression was also found to significantly correlate with surgical invasiveness of the tumor at the initial operation and may therefore be used as a potential indicator of more aggressive biological behavior.

### Predictive markers for aggressive adenoma behavior

The revision of the 2004 WHO classification defines atypical adenomas by the following histopathological criteria: MIB-1 proliferative index of >3%, elevated mitotic index, and extensive nuclear staining for p53 [30].

MIB-1 labelling index, the IHC staining of the cell cycle specific antigen Ki-67, has been shown to correlate with an increased growth rate and invasive growth [21, 31, 32]. Because a uniform correlation could not be found in all studies, it was suggested that MIB-1 alone has limited prognostic value to predict recurrence [33]. Additionally, an overlap of the MIB-1 labelling index was found between fast [<2 years tumor volume doubling time (TVDT)] and slow (≥2 years TVDT) growing pituitary adenomas [34].

The current WHO classification does not include a clear cut-off for p53 or mitosis. Trouillas et al. proposed certain values in a French multicenter case–control study [26]. However, not all studies have found a correlation of excessive p53 immunoreactivity with increased proliferation and recurrence [35, 36].

In sum, the established markers only have limited value for prediction of aggressive adenoma behavior and alternative markers are therefore needed. In a previous study on non-functioning adenomas as well as based on our results from the current study we could show that low-to-moderate MGMT immunoexpression correlates with early recurrence [27] and with invasiveness and recurrence in functioning pituitary adenomas which has been described by other authors [37–39]. Furthermore, in logistic regression analyses of all evaluated markers, low-to-moderate MGMT immunoexpression was the only variable that remained independently significant for predicting invasiveness. However, invasiveness does not necessarily correlate with more aggressive behavior, as we have found 6 cases (16%) with signs of intraoperative invasiveness in the group ER.

Low MGMT immunoexpression potentially increases mutagenesis, which may cause tumor formation and an increased cellular proliferation rate [40]. We therefore suggest evaluation of MGMT status as an additional marker to MIB-1 (as it still the only quantifiable marker adopted from the WHO) for predicting aggressive biological behavior.

### Stratification of MGMT immunoexpression

To date, MGMT immunoexpression has been assessed in pituitary adenomas for prediction of the response to TMZ treatment [41]. Differences in methodology of MGMT IHC and age of fixation (which may give false low MGMT immunoexpression) are present so that a positive internal control must be present to report low MGMT immunoexpression [15, 41, 42]. Therefore, MGMT should be performed in expert centers.

Furthermore, there has not been an agreement on the optimal stratification of MGMT immunoexpression by IHC in the literature [10, 12, 37, 42, 43]. Most authors agree that a negative or low immunoexpression should be defined as <10% of positively stained cells. Our data in functioning pituitary adenomas, taken together with the findings by Widhalm et al. in non-functioning adenomas, may suggest that MGMT expression <50% (low-to-moderate) could identify tumors with the potential for more aggressive biological behavior [27]. A recent large series of aggressive pituitary adenomas treated with TMZ reported a positive treatment response with the same cut-off value [19]. We therefore suggest including MGMT IHC in routine pituitary IHC analysis.

### Effect of radiation on MGMT immunoexpression

It is not clear whether MGMT immunoexpression is affected by radiation [12, 43–46]. Our results are in line with the literature that previous treatment and radiation increases MGMT immunoexpression [47]. In our series, MGMT immunoexpression changed after radiation therapy from low (<10%) to moderate/high (>10%) in 3/4 cases (75%) 2 ACTH and 1 PRL adenoma. In the remaining case (1 ACTH), MGMT immunoexpression remained stable. GH adenomas did not necessitate any further surgery so that tumor samples were not available.A change of MGMT immunoexpression pattern may be due to selection of more radio-resistant cell clones as shown in different cell lineages [48]. Therefore, we suggest that MGMT immunoexpression should be reassessed at post-irradiation surgery.

### Prognostic value of MSH6

The MSH6 protein plays an important role in the DNA mismatch repair (MMR) pathway. This pathway is involved in the removal of DNA base mismatches caused either by errors in DNA replication or by DNA damage [49, 50]. Loss of DNA MMR, was found to facilitate the occurrence of mutations in genes controlling proliferation and/or apoptosis thus leading to an increased risk of cancer (Lynch syndrome) [51].

Deficiency in MSH6 has been found to cause resistance to TMZ and tumor progression irrespective of MGMT status in glioblastoma and in aggressive pituitary adenomas or carcinomas [16, 18, 51–53].

To define the prognostic value of MSH6 deficiency for prediction of recurrence and invasiveness in pituitary adenomas, we evaluated the MSH6 status in the present series. We found no difference in MSH6 immunonegativity between recurrent cases and patients in remission. However, MSH6 immunoexpression did correlate with invasiveness, but failed to do so in a logistic regression.

### Limitations

The main limitation of the presented study is due to the inherent character of retrospective analysis, namely, selection bias, observer bias and data integrity.Although our single center data of functioning pituitary macroadenomas permitted us to fulfill the criteria of approximately equal follow-up period, patient age and gender distribution between the groups, an equal distribution of tumor size could not be reached because with larger tumor size, the likelihood of invasion rises [54, 55]. The situation is similar with GH adenoma granulation pattern, as sparsely granulated types tend to show a larger tumor size and more frequent signs of invasiveness than densely granulated adenomas [56]. This might confound the interpretation of our study findings.

## Conclusion

In our series, low-to-moderate MGMT immunoexpression was the only marker that significantly correlated with surgical invasiveness and recurrence in functioning pituitary macroadenomas. In the future, MGMT status may therefore be considered to be an additional marker for understanding the biological behavior of pituitary adenomas.
